# Circular RNAs’ cap-independent translation protein and its roles in carcinomas

**DOI:** 10.1186/s12943-021-01417-4

**Published:** 2021-09-15

**Authors:** Lian He, Changfeng Man, Shouyan Xiang, Lin Yao, Xiaoyan Wang, Yu Fan

**Affiliations:** 1 Cancer Institue, Affiliated People’s Hospital of Jiangsu University, No 8, Dianli Road, Zhenjiang, Jiangsu Province 212002 People’s Republic of China; 2 Department of Gastroenterology, Affiliated Suqian First People’s Hospital of Nanjing Medical University, No 120, Suzhi Road, Suqian, Jiangsu Province 223812 People’s Republic of China

**Keywords:** Carcinoma, Circular RNA, Translation, CircRNA-derived protein

## Abstract

Circular RNAs a kind of covalently closed RNA and widely expressed in eukaryotes. CircRNAs are involved in a variety of physiological and pathological processes, but their regulatory mechanisms are not fully understood. Given the development of the RNA deep-sequencing technology and the improvement of algorithms, some CircRNAs are discovered to encode proteins through the cap-independent mechanism and participate in the important process of tumorigenesis and development. Based on an overview of CircRNAs, this paper summarizes its translation mechanism and research methods, and reviews the research progress of CircRNAs translation in the field of oncology in recent years. Moreover, this paper aims to provide new ideas for tumor diagnosis and treatment through CircRNAs translation.

## Introduction

According to the latest global cancer burden data released by the International Agency for Research on Cancer of the World Health Organization in 2020, 19.29 million new cancer cases and 9.96 million deaths are observed worldwide in 2020. Malignant tumors seriously threaten people’s health. Advances in biotechnology in recent years have deepened our understanding of the complex genetic and non-genetic heterogeneities within individual tumors. The combination of proteomics and genomics may reveal the most accurate information about the activity status of a single gene. Molecular diagnosis and treatment can help improve the poor prognosis of malignant tumors [1]. Therefore, the molecular mechanism of the occurrence and development of malignant tumors should be deeply explored.

CircRNAs are a type of single-stranded covalently closed RNA molecule, and produced by non-canonical reverse splicing events [2]. CircRNAs are important in the occurrence and development of human malignant tumors [3, 4]. Given the lack of typical mRNA characteristics, CircRNAs are generally considered to be a subtype of non-coding RNA (ncRNA) [5, 6]. However, some characteristics of CircRNAs suggest translational potential. For example, most CircRNAs are composed of exon sequences, are mainly located in the cytoplasm, and can even carry a translatable open reading frame (ORF) containing a start codon. Therefore, since the 1970s, scholars devoted to reveal the translation potential of CircRNAs. Early reports indicated that most CircRNAs are not related to multimers [7]. Later, studies show that CircRNAs can be used as translation templates in viruses [8]. A study in 1995 showed that the synthetic circular RNA can recruit 40S ribosomal subunits and translate detectable polypeptides in human cells through internal entry sites [9]. Some subsequent studies focused on the endogenous CircRNA molecules, but failed to find evidence to support the translation of CircRNAs in cells [6, 10, 11], but the research is continuing. In recent years, some studies provided strong evidence for the translation of CircRNAs in cells [12–14]. New studies also questioned the translation capabilities of CircRNAs. Stagsted et al. identified and characterized a new subset of CircRNAs, i.e., AUG circRNAs, encompassing the annotated translational start codon from protein-coding host genes. Thorough cross-species analysis, extensive ribosome analysis, proteomics analysis and related experimental data on a selected group of AUG circRNAs, showed CircRNAs have no sign of translation [15]. The translation of CircRNAs is still a controversial issue, and is worthy of further exploration. Recently, more studies based on ribosome maps and improved mass spectrometry showed that some CircRNAs have translation functions. CircRNAs can achieve cap-independent translation through the internal ribosome entry site (IRES), N6-methyladenosine (m6A), or a unique rolling circle amplification (RCA) method, and the protein produced by CircRNAs may be involved in the occurrence of tumors. CircRNAs play an important biological function in development. The translation function of CircRNAs has remarkably enriched genomics and proteomics and provides a new perspective for tumor diagnosis and treatment to improve status quo.

## CircRNA overview

**Fig. 1:**
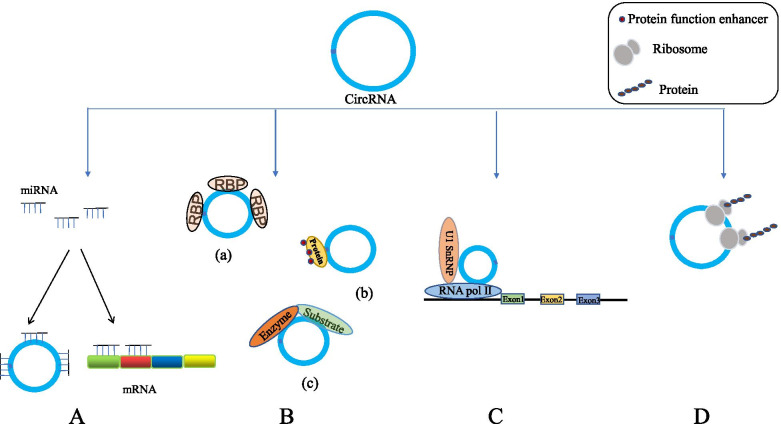
The function of circular RNA. **A** CircRNAs act as a sponge of miRNAs and play the role of CeRNA, thereby regulating the expression of mRNA. **B** CircRNAs interact with proteins as follows: a. CircRNAs act as RBP sponges; b. CircRNAs bind to certain proteins to enhance their functions; c. CircRNAs act as protein scaffolds to promote the co-localization of enzymes and their substrates; **C** CircRNAs pass Combines with RNA pol II and U1 snRNP to promote the transcription of parental genes. **D** CircRNAs translate into proteins

CircRNAs are a type of RNA with unique structure and unknown function. In 1976, Sanger et al. first discovered CircRNAs in RNA viruses [16]. In 1979, Hsu et al. observed CircRNAs in eukaryotic cells by using an electron microscope [17]. For the biosynthesis of CircRNAs, two models, namely, “exon skipping” and “direct reverse splicing circularization”, has been proposed. Both methods involve mechanically catalyzed reverse splicing of the spliceosome [18, 19]. The closed-loop structure formed by reverse splicing results in the lack of cap structure and Poly A tail in CircRNAs and is more resistant to exonuclease degradation compared with linear RNAs [10]. In accordance with the origin of the genome and way of splicing, CircRNAs are usually divided into three types, i.e.,intron CircRNAs (CiRNAs) [20], exon CircRNAs (EcircRNAs) [21] and exon–intron CircRNAs (EIciRNAs) [22]. Recently, some new forms of CircRNAs including fusion CircRNAs (f-circRNAs) [23, 24], read-through CircRNAs (rt-CircRNAs) [25, 26] and CircRNAs derived from mitochondrial DNA (mecciRNAs), have been reported [27] (Table 1). CircRNAs containing introns are isolated in the nucleus, whereas EcircRNAs are exported to the cytoplasm or exosomes [28, 29]. Initially, CircRNAs are considered to be meaningless, low-abundance splicing byproducts [30]. Given the development of sequencing technology and bioinformatics, CircRNAs can be produced by thousands of genes, and the same gene can also produce different CircRNAs through alternating cycles [10, 31]. Many CircRNAs are found in all known eukaryotes, and their expression in different species is often highly conserved [32, 33]. Increasing studies showed that CircRNA plays an important role in the occurrence, development and prognosis of various diseases especially tumors [34, 35] (Table 2). In addition, CircRNAs is related to the process of neurodevelopment [36], autoimmune response [37] and infertility [38]. Considering their abundance, high stability, tissue-specific expression pattern and wide distribution in various body fluids, CircRNAs have remarkable potential as a biomarker for the liquid biopsy of human diseases [39–41]. Currently known CircRNAs can act as miRNA sponges [5, 42, 43] (Fig. 1a) or interact with proteins [44] (Fig. 1b) and can regulate the expression of parental genes [22] (Fig. 1c) and participate in the occurrence and development of the disease. Recently, some scholars discovered that some CircRNAs can be effectively translated into detectable peptides and play a regulatory role in disease. Translation is the hidden function of CircRNAs, and its underlying mechanism is still unclear and requires in-depth exploration to a large extent.

**Table 1:** Different types of circRNAs

**Categories**	**Composition characteristics**	**Main positioning**	**The main function**	**Ref.**
**CiRNAs**	Intron splicing	Nucleus	Participate in regulating the expression of its parental genes	[20]
**EcircRNAs**	Exon splicing	Cytoplasm	As a sponge of miRNA or interact with RBPs	[34]
**EIciRNAs**	Contains introns and exons	Nucleus	Promote the transcription of its host gene by interacting with U1 small nuclear ribonucleoprotein (snRNP)	[22]
**f−circRNAs**	Linear fusion transcript derived from genome rearrangement (chromosomal translocation)	Cytoplasm and nucleus	Contribute to cell transformation, promote cell viability and drug resistance after treatment, and have tumor-promoting properties in in vivo models	[23]
**rt− circRNAs**	Coding exons of two adjacent and similarly oriented genes (read-through transcription)	Cytoplasm	To be elucidated, it may be a mechanism of gene regulation in a specific environment; it may be a mechanism of protein complex evolution	[26]
**mecciRNAs**	Mitochondrial Genome	Mitochondria inside and outside	As a molecular chaperone, it helps to fold nuclear-encoded proteins and facilitate their entry into the mitochondria.	[45]

**Table 2:** Dysregulated circRNAs in cancer

**CircRNA**	**Cancer type**	**Mechanism**	**Biological function**	**Ref**
CircMEMO1	hepatocellular carcinoma(HCC)	As a sponge for miR-106b-5p	Inhibit cancer cell proliferation, migration and invasion and increase the sensitivity of HCC cells to sorafenib treatment.	[46]
CircMALAT1	hepatocellular carcinoma	Acts as mRNA translation brake and miR-6887-3p sponge	Promote the self-renewal of hepatocellular carcinoma stem cells	[47]
CircIGHG	oral squamous cell carcinoma	Acts as miR-142-5p sponge	Promote cancer cell invasiveness	[48]
CircMRPS35	gastric cancer	Acts as a modular scaffold to recruit histone acetyltransferase KAT7	Inhibit the proliferation and invasion of cancer cells	[49]
CircACTN4	breast cancer	Binding far upstream element binding protein 1	Promotes cancer cell proliferation, invasion and migration	[50]
CircSKA3	breast cancer	Binds integrin β1 to induce Invadopodium formation	Promote cancer cell migration and invasion	[51]
CircNTRK2	Esophageal cancer	Acts as a sponge for miR-140-3p	Promotes cancer cell proliferation, invasion and migration	[52]
CircNDUFB2	non-small cell lung cancer (NSCLC)	As a scaffold to enhance the interaction between TRIM25 and IGF2BPs	A suppressor in the progression of NSCLC	[53]
CircSDHC	renal cell carcinoma(RCC)	Serves as a sponge for miR-127-3p	promotes RCC proliferation and aggression	[54]
CircPTPRA	bladder cancer	Interacting with IGF2BP1	A tumor suppresso	[55]
CircZFR	cervical cancer	Bounding with SSBP1 to promote the assembly of CDK2/cyclin E1 complexes	Promotes cancer cell proliferation, invasion and migration	[55]
Circ2082	glioblastoma	Binds to RMB3 and is part of the nuclear DICER complex	Reduce the tumorigenicity of glioblastoma cells	[56]
CircECE1	osteosarcoma	Interacts with c-Myc to prevent speckle-type POZ-mediated c-Myc ubiquitination and degradation	Inhibit cancer cell proliferation and metastasis	[57]
CircBFAR	Pancreatic cancer	Sponge miR-34b-5p up-regulates the expression of mesenchymal-epithelial transition factor	Promotes cancer cell proliferation, invasion and migration	[58]
CircSPARC	Colorectal cancer	Sponge miR-485-3p to upregulate JAK2 expression	Enhances tumour growth and metastasis	[59]
Circ_0020710	melanoma	Sponge miR-370-3p to up-regulate the expression of CXCL12	Promotes cancer cell proliferation, invasion and migration	[60]
CircCRIM1	nasopharyngeal carcinoma	Sponge miR-422a to block the suppression effect of miR-422a on FOXQ1	Promote cancer cell migration and invasion	[61]
CircTNPO3	Ovarian Cancer	Sponge miR-1299 to up-regulate NEK2 expression	Enhance the resistance of ovarian cancer cells to PTX	[62]
Circ_0002577	endometrial cancer	Acts as a miR-625-5p sponge, upregulating IGF1R and activating the PI3K/Akt pathway	Promotes cancer cell proliferation, invasion and migration	[63]

## CircRNA translation mechanism

Generally speaking, the translation of RNA in eukaryotic cells requires the eukaryotic translation initiation factor eIF4F. eIF4F is a complex composed of helicase eIF4A, cap-binding subunit eIF4E, and scaffold protein eIF4G. eIF4G can attract the 43S pre-initiation complex (PIC, loaded with eIF1, eIF1A, eIF3, eIF5 and the 40 s subunit of the ternary complex eIF2xMet–tRNAiMetx GTP) by interacting with eIF3 [64, 65]. When eIF4F recognizes the 5′-end 7-methylguanosine (m7G) cap structure of mRNA and recruits the 43S complex, the translation process begins. This mechanism is called the the cap-dependent translation pathway [66, 67], and the main mechanism of eukaryotic translation initiation, but is not the only mechanism. Under certain circumstances, such as stress, a cap-independent translation mechanism is present in the body [68–70]. The cap-independent translation pathway is the underlying mechanism of CircRNA translation.

### IRES

**Fig. 2:**
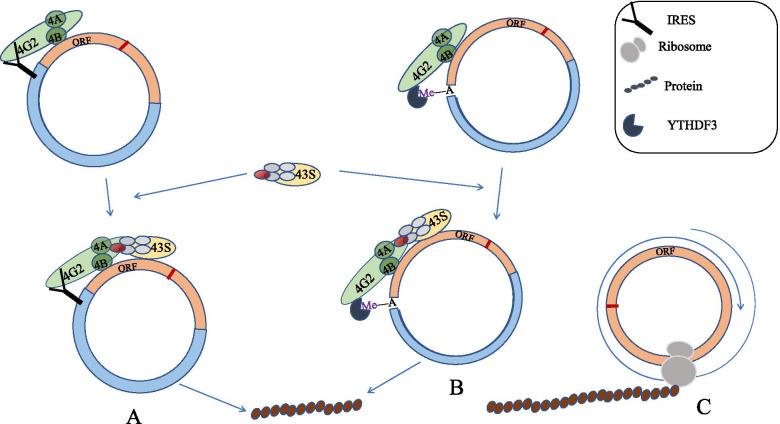
CircRNAs translation mechanism. **A** The non-classical eIF4G protein (eIF4G2) directly recognizes the IRES on CircRNAs to assemble the eIF4 complex to translate the downstream ORF. **B** YTHDF3 can recognize m6A in CircRNAs and recruit eIF4G2 to m6A to initiate translation. **C** CircRNA containing infinite open reading frame (iORF) undergoes rolling circle amplification to achieve translation

The IRES, a secondary structure sequence located in the non-coding region of the 5’-end of mRNA [71], can directly recruit ribosomes to initiate translation and is a cap-independent translation. The IRES-mediated translation is first discovered in viruses [72, 73], and cellular IRES lacks sequential/structural similarity and appears to be richer and more complex compared with viral IRES. The IRES-mediated translation in eukaryotic cells can serve as an emergency maintenance mechanism to ensure that the body’s basic protein requirements are met during stress [74, 75]. This mechanism is likely to be triggered by viral invasion, tumors or other diseases [76, 77]. The high-throughput screening of IRES elements in the human genome shows that about 10% of human mRNAs contain IRES elements [78]. In 2016, Chen et al. established CircRNADb on the basis of 32,914 human EcircRNAs. During preliminary exploration, they predicted that about half of these CircRNAs contain ORF. The VIPS method based on RNA structural similarity predicts that about half of CircRNAs containing ORFs contain IRES [79]. Other studies showed that a large number of AU-rich motifs (0—10nt) have IRES-like activity and can initiate CircRNA translation [80]. These seems to indicate the importance and universality of the IRES-mediated cap-independent translation mechanism. At present, IRES is one of the widely accepted translation initiation mechanisms of CircRNAs [14, 81]. However, knowledge about the mechanism of IRES-mediated ribosome assembly is lacking. Some studies believed that IRES can be directly recognized by the non-classical eIF4G protein (eIF4G2). Unlike eIF4G, eIF4G2 contains eIF4A- and eIF3-binding regions but does not have a binding site for the cap-binding subunit eIF4E [82, 83]. Therefore, even without the 5’-end cap structure, the IRES on CircRNAs can assemble the eIF4 complex and directly translate the downstream ORF (Fig. 2a). Functional studies showed that IRES relies on a special molecular structure. Thus the 40S subunit can be assembled on sequences other than the 5’-end [66]. A class of proteins called IRES trans-acting factors (ITAFs) assist IRES to recruit ribosomes for the initiation of translation through a variety of mechanisms [71, 84, 85].

### m6A-induced ribosome engagement site (MIRES)

m6A is the most abundant RNA internal modification in eukaryotes [86]. This modification mostly occurs in the shared motif “RRm6ACH” (R = G or A; H = A, C, or U) [87], affecting multiple stages of RNA localization, splicing, translation, and degradation [69]. m6A is catalyzed by a methyltransferase complex composed of METTL3, METL14, Wilms tumor-associated protein, RBM15/15B, Virma, and ZC3H13 [88, 89] and by cellulite and obesity-related proteins (FTO) and ALKB homolog 3/5 (ALKBH3/5) demethylated [90–92]. At the same time, YT521-B homology domain (YTH) family of proteins, including YTHDC1, YTHDC2, YTHDF1, YTHDF2 and YTHDF3 and IGF2BP1/2/3; hnRNP; and eIF3 are required as “readers” to perform specific biological functions [92, 93]. Studies showed that m6A is enriched in many CircRNA sequences and tends to appear in the upstream and middle regions of larger exons [94]. Although some CircRNAs do not have a natural IRES, a single m6A site is sufficient to initiate translation [13]. This mechanism is another cap-independent translation mechanism called the (MIRES) [95]. At present, this mechanism, remains unclear but with some understanding. Studies showed that YTHDF3 can recognize m6A and recruit eIF4G2 to m6A to initiate translation, whereas methyltransferase and demethylase can enhance and inhibit translation respectively [90, 91, 96, 97] (Fig. 2b). The deletion of YTHDF3 inhibits the translation of CircRNAs [13]. Another study showed that m6A can directly bind to EIF3 without the cap-binding subunit eIF4E, thereby recruiting the 43S pre-priming complex to initiate translation [95].

### Rolling circle amplification

By synthesizing special CircRNAs in vitro and transfecting them into eukaryotic cells, studies showed that similar to the polymerase reaction the RCA method completes the translation of CircRNAs [98]. The number of nucleotides of these CircRNAs is a multiple of three and contains the start codon AUG but no IRES or stop codon. This finding indicates that these CircRNAs contains an infinite open reading frame (iORF) [99]. Therefore, theoretically, once translation begins, the extension can rotate around the ring multiple times to produce high-molecular weight proteins (Fig. 2C). A recent study proved that the RCA of CircRNAs also exists in the body, and proposed that the programmed -1 ribosomal frameshift induced out-of-frame stop codon can terminate the iORF translation process [100]. Therefore, this single but inefficiently initiated RCA mechanism may be another mechanism of CircRNA translation [101, 102].

## Research methods of circRNA translation

The earliest report of CircRNAs as a translation template is the study of viral nucleic acid in 1986 [8]. However, the translation ability of CircRNAs has always been controversial, requires different methods to study the structural and functional characteristics of circular RNA, and clearly demonstrate its coding potential in vivo. For RNA with translation capability, ORF is essential, and long, and conservative ORFs are likely to be encoded [103, 104]. However, for CircRNAs, a short open reading frame (sORF) that is usually less than 300 nt in length seems to be important [105–107], and ORF spanning splice sites are also a significant feature of CircRNA-encoding peptides [81]. Aiming at the characteristics of CircRNAs with translation potential and their potential translation mechanism, current research ideas include reading frame prediction, translation initiation component prediction, conservative analysis, translationomics, proteomics, and many other aspects. Research methods also include the methods of bioinformatics predictive analysis and experimental verification.

Recently, specialized databases that integrate multiple methods and data are gradually developed. The RiboCIRC analyzes 3168 publicly available Ribo-seq and 1970 matched RNA-seq data sets. These data cover 314 studies of 21 different species, and established a comprehensive translatable CircRNA database that is predicted by calculations and verified by experiments [108]. The TransCirc is also recently established a comprehensive database [109]. The TransCirc contains information on more than 300,000 CircRNAs and has obtained multi-omics evidence to support CircRNA translation from published literature. Through a combination of direct and indirect evidence, The TransCirc predicts the potential of all CircRNAs in encoding functional peptides.

After calculation and evaluation, experimental methods should be used to identify the translation of CircRNAs. The Ribosome Atlas, the deep sequencing of mRNA fragments protected by ribosomes, is a powerful tool for the global monitoring of protein translation in the body. The Ribosome Atlas can discover the regulation of gene expression behind various complex biological processes, important aspects of protein synthesis mechanisms, and even new proteins [110, 111]. The sequencing of the ribosome footprint provides an accurate record of the position of the ribosome when translation stops, revealing what transcript is being translated by the ribosome. The overall density of the ribosome footprint reflects the rate of translation that occurs on different transcripts, allowing a direct and quantitative measure of the rate at which each protein is produced by the cell [112]. Polysome profiling is based on the high sedimentation coefficient of ribosomes, and the sucrose density gradient centrifugation can be used to separate polyribosomes. Analyzing the separated components can evaluate the coding potential of ncRNA and directly determine the translation efficiency of CircRNAs in the whole genome [113, 114]. Ribosome profiling and polymer separation are two complementary methods that can realize genome-wide translation analysis. Proteomics can be used to discover and directly detect the micropeptides encoded by circRNAs, and the biological mass spectrometry is a common identification and analysis method for these micropeptides [115, 116].

## CircRNA translation peptides in tumors

Thus far, identified translatable CircRNAs are lacking. For the function of the protein obtained by CircRNA translation, Zhao et al. proposed the protein bait hypothesis. The protein encoded by CircRNAs competes with its homologous linear splicing protein isoforms for binding molecule, thereby preventing the normal function of the isomer [117]. CircRNA disorders are common in tumors including basal cell tumors, and the unbalanced expression of CircRNA-encoded proteins may contribute to the occurrence and development of tumors [25]. Based on the abnormal expression pattern of CircRNAs in human malignant tumors, CircRNA-encoded proteins can provide new specific targets for tumor diagnosis and treatment.

### Glioblastoma (GBM)

At present, more coded CircRNAs are found in brain gliomas than in other tumors. This may be related to the increased CircRNAs in neuronal tissues [118]. In GBM CircFBXW7 can encode a new 21 kDa protein FBXW7-185aa through IRES [119]. CircFBXW7 and FBXW7-185aa are less expressed than adjacent tissues. The expression of CircFBXW7 is positively correlated with the overall survival of patients with GBM, whereas FBXW7-185aa induces c-myc stabilization by antagonizing USP28, leading to cell cycle arrest and inhibited proliferation of GBM cells. Circ-SHPRH is expressed in large amounts in normal human brains but low in GBM. The tandem stop codon “UGAUGA” is generated during the cyclization of Circ-SHPRH. Using this overlapping genetic code, Circ-SHPRH can start and stop translation, resulting in a new functional protein SHPRH-146aa [120]. SHPRH-146aa is a tumor suppressor that can protect the full-length SHPRH protein from being degraded by the ubiquitin proteasome, thereby promoting the turnover of proliferating cell nuclear antigen in vivo. Similar to CircSHPRH, CircAKT3, which has low expression in GBM, uses overlapping start-stop codons to encode the new protein AKT3-174aa with 174 amino acids [121]. AKT3-174aa competitively interacts with phosphorylated PDK1, reduces the phosphorylation of AKT-Thr308, and plays a negative regulatory role in regulating the PI3K/AKT signal intensity. The overexpression of AKT3174aa reduces the proliferation, radiation resistance and tumorigenicity of GBM cells. Zhang et al. also found that Circ-LINCPINT, which has low expression in GBM, encodes the 87-amino acid peptide PINT87aa [122]. The peptide can directly interact with the polymerase-related factor complex to inhibit the transcription extension of a variety of oncogenes.

In 50% of GBM, activated epidermal growth factor receptor (EGFR) signals drive tumorigenesis. Circ-E-Cad, which is highly expressed in GBM, encodes an undescribed secreted E-cadherin variant (C-E-Cad) through IRES. This protein mediates an additional mechanism to activate EGFR signaling [123]. C-E-Cad binds to the EGFR CR2 domain and activates EGFR with a unique 14-amino acid carboxyl terminus, thereby maintaining the tumorigenicity of glioma stem cells. In the treatment of GBM, the inhibition of C-E-Cad can significantly enhance the anti-tumor activity of anti-EGFR strategies. Liu et al. found that Circ-EGFR, which is up-regulated in GBM, forms a new multimeric protein complex rtEGFR through RCA and translation [100]. rtEGFR can directly interact with EGFR, maintain EGFR membrane positioning, and reduce the endocytosis and degradation of EGFR. In brain tumor-initiating cells, reducing the expression of rtEGFR weakens the tumorigenicity of tumors and enhances the effect of anti-GBM. The abnormal activation of the Hedgehog pathway is also an important factor in the occurrence of GBM. Recent studies found that the IRES-mediated Circ-SMO encodes a new protein SMO-193aa [124]. The peptide directly interacts with SMO, a key component of the Hedgehog pathway, to enhance SMO cholesterol modification, release SMO from the inhibition of patch transmembrane receptors, and activate SMO. Inhibiting the expression of SMO-193aa can weaken the Hedgehog signal strength of cancer stem cells and inhibit self-renewal, proliferation in vitro, and tumorigenicity in vivo. This finding indicates that SMO-193aa is an oncogenic protein that promotes the occurrence of GBM.

### Colon cancer

Zheng et al. found that the expression of CircPP1R12A in colon cancer tissues is significantly increased and has a small ORF (216 nt), which encodes an unidentified functional protein CircPPP1R12A-73aa [125]. The protein can promote the growth and metastasis of cancer cells by activating the Hippo-Yap signaling pathway. However, CircFNDC3B, which has expression in colon cancer cell lines and tissues, can encode a new protein CircFNDC3B-218aa through IRES [126]. CircFNDC3B-218aa but not CircFNDC3B can inhibit the proliferation, invasion, and migration of colon cancer. Further research found that CircFNDC3B-218aa, a tumor suppressor factor, inhibits the expression of Snail, thereby up-regulating FBP1 and inhibiting the epithelial to mesenchymal transition. Zhi et al. found that a high expression of CircLgr4 in colon tumors can encode polypeptides. CircLgr4-derived peptides interact with and activate LGR4, which further promotes the activation of Wnt/β-catenin signals and drives the self-renewal of colorectal cancer stem cells [127].

### Breast cancer

As mentioned earlier, CircFBXW7 encoding FBXW7-185aa has an anti-tumor effect in gliomas. Ye et al. found that in triple-negative breast cancer (TNBC), the low expression of CircFBXW can also encode the FBXW7-185aa protein. This protein inhibits the proliferation and migration of TNBC cells by increasing the abundance of FBXW7 and inducing the degradation of c-myc [128]. Li et al. proposed that about 30% of TNBC clinical specimens express a splice variant of the HER2 gene, i.e., CircHER2. Thus, some TNBCs are not truly “HER2-negative”. CircHER2 can encode a new protein HER2-103 that is identical to most of the amino acids in the CR I region of HER2 [129]. This protein can promote EGFR/HER3 homo/heterodimerization, sustained AKT phosphorylation, and downstream malignant phenotype. Pertuzumab can significantly reduce the tumorigenicity of CircHER2/HER2-103-positive TNBC cells in vivo, but has no significant effect on CircHER2/HER2-103-negative TNBC cells.

### Hepatocellular carcinoma(HCC)

CircARHGAP35 from exons 2–3 of the ARHGAP35 gene is highly expressed in HCC tissues. Studies found that CircARHGAP35 has a cancer-promoting effect in HCC, whereas the parent gene ARHGAP35 of CircARHGAP35 has a cancer-suppressing effect. Further exploration of the mechanism found that the CircARHGAP35 sequence contains a 3867 nt long ORF sequence spanning the splice junction, which can encode a new protein of 1289aa [130]. This protein overlaps with the sequence of the ARHGAP35 parent gene protein at the N-terminus and only a short segment at the C-terminus is a unique sequence. This protein also has 4 FF domains, but lacks the RhoGAP domain. The protein can be located in the nucleus and interact with TFII-I to promote the proliferation and migration of HCC cells. Circβ-catenin is also highly expressed in HCC tissues, and using the same start codon as linear β-catenin mRNA, new stop codons are formed by circularization and IRES in the sequence to achieve translation, which can generate a new 370 amino acid β-catenin subtype, i.e., β-catenin-370aa [131]. β-catenin-370aa can competitively interact with glycogen synthase kinase 3β (Gsk3β) to prevent Gsk3β from binding to full-length β-catenin. This competitive effect inhibits the phosphorylation and degradation of β-catenin induced by Gsk3β, stabilizes the full-length β-catenin, and activates the Wnt signaling pathway. The activation of Wnt pathway is related to the occurrence and poor prognosis of liver cancer.

### Gastric cancer

The down-regulated CircMAPK1 expression in gastric cancer tissue can inhibit the proliferation and invasion of gastric cancer cells in vivo and in vitro. Studies found that CircMAPK1 can encode the new protein MAPK1-109aa with 109 amino acids through IRES [132]. Functional experiments confirmed that CircMAPK1 plays a tumor-inhibiting effect through MAPK1-109aa. In terms of mechanism, the tumor suppressor MAPK1-109aa can competitively bind to MEK1 to inhibit the phosphorylation of MAPK1, thereby inhibiting the activation of MAPK1 and its downstream factors in the MAPK pathway. The MAPK cascade signaling pathway is a key signaling pathway that regulates cell proliferation, survival, and differentiation and is related to the occurrence of a variety of tumors [133].

## Conclusion and outlook

CircRNAs achieve protein translation through a cap-independent translation mechanism, which blurs the definition between coding RNA and ncRNA, and makes us realize that the translation mechanism in eukaryotic cells is much more complicated than we understand. Most of the CircRNA-encoded proteins found in most studies play an anti-tumor or tumor-promoting role through different signal transduction pathways. This finding indicates the importance of CircRNA-encoded protein in tumorigenesis and development and fully proves its potential development and clinical application values. Given the deepening theoretical and clinical research, CircRNAs and its encoded protein may be applied to tumor diagnosis and treatment through a variety of ideas. Examples include using these peptides/proteins with classic anti-cancer drugs, vaccinating synthetic peptides or viral vector vaccines encoding related peptide sequences, and developing new drugs on the basis of potential targets in the mechanism. However, most CircrNAs seem to not be related to ribosomes. Moreover, the efficiency of cap-independent translation initiation is low, and the abundance of polypeptides translated by CircrNAs is limited. At present, the research on CircRNA translation is still in its infancy. Many issues, such as refined mechanism, regulation, extension, and termination process, are unknown, and further research and exploration are needed.

## Data Availability

Not applicable.

## Abbreviations

mRNA: Messenger RNA
mecciRNAs: Mitochondria-encoded
TRIM25: The tripartite motif protein 25
KAT7: Histone acetyltransferase 7
IGF2BPs: Insulin-like growth factor 2 mRNA-binding proteins
IGF2BP1: Insulin-like growth factor 2 mRNA-binding protein1
CDK2: Cyclin-dependent kinase 2
JAK2: Janus kinase 2
CXCL12: Chemokine 12
FOXQ1: Forkhead box q1
NEK2: Never in mitosis gene A-related kinase 2
PTX: Paclitaxel
IGF1R: Insulin-like growth factor 1 receptors
PI3K: Phosphoinositide 3-kinase
eIF4F: The eukaryotic translation initiation factor 4F
eIF4A: The eukaryotic translation initiation factor 4A
eIF4E: The eukaryotic translation initiation factor 4E
eIF4G: The eukaryotic translation initiation factor 4G
eIF1: The eukaryotic translation initiation factor 1
eIF1A: The eukaryotic translation initiation factor 1A
eIF3: The eukaryotic translation initiation factor 3
eIF5: The eukaryotic translation initiation factor5
METTL3: Methyltransferase-like3
METL14: Methyltransferaselike-14
RBM15/15B: RNA-binding motif protein 15/15B
ZC3H13: Zinc finger CCCH-type containing 13
YTHDC1: YTH domain containing 1
YTHDC2: YTH domain containing 2
YTHDF1: YTH domain family protein 1
YTHDF2: YTH domain family protein 2
YTHDF3: YTH domain family protein3
hnRNP: Heterogeneous nuclear ribonucleoproteins
PDK1: The 3-phosphoinositide-dependent protein kinase 1
PAF1c: The Arabidopsis polymerase II-associated factor 1 complex
SMO: The G protein-coupled-like receptor smoothened
FBP1: The gluconeogenic enzyme fructose 1,6-bisphosphatase 1
MAPK: Mitogen-activated protein kinases

## References

[1] Bhujwalla Z, Kakkad S, Chen Z, Jin J, Hapuarachchige S, Artemov D (2018). Theranostics and metabolotheranostics for precision medicine in oncology. J Magn Reson (San Diego, Calif: 1997).

[2] Xiao MS, Ai Y, Wilusz JE (2020). Biogenesis and functions of circular RNAs come into focus. Trends Cell Biol.

[3] Qu S, Liu Z, Yang X, Zhou J, Yu H, Zhang R (2018). The emerging functions and roles of circular RNAs in cancer. Cancer Lett.

[4] Wang Y, Mo Y, Gong Z, Yang X, Yang M, Zhang S (2017). Circular RNAs in human cancer. Mol Cancer.

[5] Memczak S, Jens M, Elefsinioti A, Torti F, Krueger J, Rybak A (2013). Circular RNAs are a large class of animal RNAs with regulatory potency. Nature.

[6] Guo JU, Agarwal V, Guo H, Bartel DP (2014). Expanded identification and characterization of mammalian circular RNAs. Genome Biol.

[7] Kozak M (1979). Inability of circular mRNA to attach to eukaryotic ribosomes. Nature.

[8] Kos A, Dijkema R, Arnberg AC, van der Meide PH, Schellekens H (1986). The hepatitis delta (delta) virus possesses a circular RNA. Nature.

[9] Chen CY, Sarnow P (1995). Initiation of protein synthesis by the eukaryotic translational apparatus on circular RNAs. Science.

[10] Jeck WR, Sorrentino JA, Wang K, Slevin MK, Burd CE, Liu J (2013). Circular RNAs are abundant, conserved, and associated with ALU repeats. RNA.

[11] Schneider T, Hung LH, Schreiner S, Starke S, Eckhof H, Rossbach O (2016). CircRNA-protein complexes: IMP3 protein component defines subfamily of circRNPs. Sci Rep.

[12] Legnini I, Di Timoteo G, Rossi F, Morlando M, Briganti F, Sthandier O (2017). Circ-ZNF609 is a circular RNA that can be translated and functions in myogenesis. Mol Cell.

[13] Yang Y, Fan X, Mao M, Song X, Wu P, Zhang Y (2017). Extensive translation of circular RNAs driven by N(6)-methyladenosine. Cell Res.

[14] Pamudurti NR, Bartok O, Jens M, Ashwal-Fluss R, Stottmeister C, Ruhe L (2017). Translation of CircRNAs. Mol Cell.

[15] Stagsted LV, Nielsen KM, Daugaard I, Hansen TB (2019). Noncoding AUG circRNAs constitute an abundant and conserved subclass of circles. Life Sci Alliance.

[16] Sanger HL, Klotz G, Riesner D, Gross HJ, Kleinschmidt AK (1976). Viroids are single-stranded covalently closed circular RNA molecules existing as highly base-paired rod-like structures. Proc Natl Acad Sci U S A.

[17] Hsu MT, Coca-Prados M (1979). Electron microscopic evidence for the circular form of RNA in the cytoplasm of eukaryotic cells. Nature.

[18] Ashwal-Fluss R, Meyer M, Pamudurti NR, Ivanov A, Bartok O, Hanan M (2014). circRNA biogenesis competes with pre-mRNA splicing. Mol Cell.

[19] Jeck WR, Sharpless NE (2014). Detecting and characterizing circular RNAs. Nat Biotechnol.

[20] Zhang Y, Zhang XO, Chen T, Xiang JF, Yin QF, Xing YH (2013). Circular intronic long noncoding RNAs. Mol Cell.

[21] Chen N, Zhao G, Yan X, Lv Z, Yin H, Zhang S (2018). A novel FLI1 exonic circular RNA promotes metastasis in breast cancer by coordinately regulating TET1 and DNMT1. Genome Biol.

[22] Li Z, Huang C, Bao C, Chen L, Lin M, Wang X (2015). Exon-intron circular RNAs regulate transcription in the nucleus. Nat Struct Mol Biol.

[23] Guarnerio J, Bezzi M, Jeong JC, Paffenholz SV, Berry K, Naldini MM (2016). Oncogenic role of fusion-circRNAs derived from cancer-associated chromosomal translocations. Cell.

[24] Tan S, Sun D, Pu W, Gou Q, Guo C, Gong Y (2018). Circular RNA F-circEA-2a derived from EML4-ALK fusion gene promotes cell migration and invasion in non-small cell lung cancer. Mol Cancer.

[25] Vo JN, Cieslik M, Zhang Y, Shukla S, Xiao L, Zhang Y (2019). The landscape of circular RNA in cancer. Cell.

[26] Vidal AF (2020). Read-through circular RNAs reveal the plasticity of RNA processing mechanisms in human cells. RNA Biol.

[27] Liu X, Wang X, Li J, Hu S, Deng Y, Yin H (2020). Identification of mecciRNAs and their roles in the mitochondrial entry of proteins. Sci China Life Sci.

[28] Ledford H (2013). Circular RNAs throw genetics for a loop. Nature.

[29] Chen I, Chen CY, Chuang TJ (2015). Biogenesis, identification, and function of exonic circular RNAs. Wiley Interdiscip Rev RNA.

[30] Nigro JM, Cho KR, Fearon ER, Kern SE, Ruppert JM, Oliner JD (1991). Scrambled exons. Cell.

[31] Salzman J, Gawad C, Wang PL, Lacayo N, Brown PO (2012). Circular RNAs are the predominant transcript isoform from hundreds of human genes in diverse cell types. PLoS One.

[32] Salzman J, Chen RE, Olsen MN, Wang PL, Brown PO (2013). Cell-type specific features of circular RNA expression. PLoS Genet.

[33] Ye CY, Chen L, Liu C, Zhu QH, Fan L (2015). Widespread noncoding circular RNAs in plants. New Phytol.

[34] Shang Q, Yang Z, Jia R, Ge S (2019). The novel roles of circRNAs in human cancer. Mol Cancer.

[35] Meng S, Zhou H, Feng Z, Xu Z, Tang Y, Li P (2017). CircRNA: functions and properties of a novel potential biomarker for cancer. Mol Cancer.

[36] Dube U, Del-Aguila JL, Li Z, Budde JP, Jiang S, Hsu S (2019). An atlas of cortical circular RNA expression in Alzheimer disease brains demonstrates clinical and pathological associations. Nat Neurosci.

[37] Cardamone G, Paraboschi EM, Rimoldi V, Duga S, Soldà G, Asselta R (2017). The characterization of GSDMB splicing and backsplicing profiles identifies novel isoforms and a circular RNA that are dysregulated in multiple sclerosis. Int J Mol Sci.

[38] Shen J, Chen L, Cheng J, Jin X, Mu Y, Li Q (2019). Circular RNA sequencing reveals the molecular mechanism of the effects of acupuncture and moxibustion on endometrial receptivity in patients undergoing infertility treatment. Mol Med Rep.

[39] Lei B, Tian Z, Fan W, Ni B (2019). Circular RNA: a novel biomarker and therapeutic target for human cancers. Int J Med Sci.

[40] Beilerli A, Gareev I, Beylerli O, Yang G, Pavlov V, Aliev G, et al. Circular RNAs as biomarkers and therapeutic targets in cancer. Semin Cancer Biol. 2021. 10.1016/j.semcancer.2020.12.026. 10.1016/j.semcancer.2020.12.026 33434640

[41] Wen G, Zhou T, Gu W. The potential of using blood circular RNA as liquid biopsy biomarker for human diseases. Protein Cell. 2020. 10.1007/s13238-020-00799-3. 10.1007/s13238-020-00799-3 PMC8674396 33131025

[42] Hansen TB, Jensen TI, Clausen BH, Bramsen JB, Finsen B, Damgaard CK (2013). Natural RNA circles function as efficient microRNA sponges. Nature.

[43] Jens M, Rajewsky N (2015). Competition between target sites of regulators shapes post-transcriptional gene regulation. Nat Rev Genet.

[44] Abdelmohsen K, Panda AC, Munk R, Grammatikakis I, Dudekula DB, De S (2017). Identification of HuR target circular RNAs uncovers suppression of PABPN1 translation by CircPABPN1. RNA Biol.

[45] Liu X, Yang Y, Shan G. Identification and detection of mecciRNAs. Methods. 2021;S1046–2023(21):00043–8. 10.1016/j.ymeth.2021.02.006 33588027

[46] Dong ZR, Ke AW, Li T, Cai JB, Yang YF, Zhou W (2021). CircMEMO1 modulates the promoter methylation and expression of TCF21 to regulate hepatocellular carcinoma progression and sorafenib treatment sensitivity. Mol Cancer.

[47] Chen L, Kong R, Wu C, Wang S, Liu Z, Liu S (2020). Circ-MALAT1 functions as both an mRNA translation brake and a microRNA sponge to promote self-renewal of hepatocellular cancer stem cells. Adv Sci (Weinh).

[48] Liu J, Jiang X, Zou A, Mai Z, Huang Z, Sun L (2021). circIGHG-induced epithelial-to-mesenchymal transition promotes oral squamous cell carcinoma progression via miR-142-5p/IGF2BP3 signaling. Cancer Res.

[49] Jie M, Wu Y, Gao M, Li X, Liu C, Ouyang Q (2020). CircMRPS35 suppresses gastric cancer progression via recruiting KAT7 to govern histone modification. Mol Cancer.

[50] Wang X, Xing L, Yang R, Chen H, Wang M, Jiang R (2021). The circACTN4 interacts with FUBP1 to promote tumorigenesis and progression of breast cancer by regulating the expression of proto-oncogene MYC. Mol Cancer.

[51] Du WW, Yang W, Li X, Fang L, Wu N, Li F (2020). The circular RNA circSKA3 binds integrin β1 to induce invadopodium formation enhancing breast cancer invasion. Mol Ther.

[52] Chen X, Jiang J, Zhao Y, Wang X, Zhang C, Zhuan L (2020). Circular RNA circNTRK2 facilitates the progression of esophageal squamous cell carcinoma through up-regulating NRIP1 expression via miR-140-3p. J Exp Clin Cancer Res.

[53] Li B, Zhu L, Lu C, Wang C, Wang H, Jin H (2021). circNDUFB2 inhibits non-small cell lung cancer progression via destabilizing IGF2BPs and activating anti-tumor immunity. Nat Commun.

[54] Cen J, Liang Y, Huang Y, Pan Y, Shu G, Zheng Z (2021). Circular RNA circSDHC serves as a sponge for miR-127-3p to promote the proliferation and metastasis of renal cell carcinoma via the CDKN3/E2F1 axis. Mol Cancer.

[55] Xie F, Huang C, Liu F, Zhang H, Xiao X, Sun J (2021). CircPTPRA blocks the recognition of RNA N(6)-methyladenosine through interacting with IGF2BP1 to suppress bladder cancer progression. Mol Cancer.

[56] Zhou M, Yang Z, Wang D, Chen P, Zhang Y (2021). The circular RNA circZFR phosphorylates Rb promoting cervical cancer progression by regulating the SSBP1/CDK2/cyclin E1 complex. J Exp Clin Cancer Res.

[57] Bronisz A, Rooj AK, Krawczyński K, Peruzzi P, Salińska E, Nakano I (2020). The nuclear DICER-circular RNA complex drives the deregulation of the glioblastoma cell microRNAome. Sci Adv.

[58] Guo X, Zhou Q, Su D, Luo Y, Fu Z, Huang L (2020). Circular RNA circBFAR promotes the progression of pancreatic ductal adenocarcinoma via the miR-34b-5p/MET/Akt axis. Mol Cancer.

[59] Wang J, Zhang Y, Song H, Yin H, Jiang T, Xu Y (2021). The circular RNA circSPARC enhances the migration and proliferation of colorectal cancer by regulating the JAK/STAT pathway. Mol Cancer.

[60] Wei CY, Zhu MX, Lu NH, Liu JQ, Yang YW, Zhang Y (2020). Circular RNA circ_0020710 drives tumor progression and immune evasion by regulating the miR-370-3p/CXCL12 axis in melanoma. Mol Cancer.

[61] Hong X, Liu N, Liang Y, He Q, Yang X, Lei Y (2020). Circular RNA CRIM1 functions as a ceRNA to promote nasopharyngeal carcinoma metastasis and docetaxel chemoresistance through upregulating FOXQ1. Mol Cancer.

[62] Xia B, Zhao Z, Wu Y, Wang Y, Zhao Y, Wang J (2020). Circular RNA circTNPO3 regulates paclitaxel resistance of ovarian cancer cells by miR-1299/NEK2 signaling pathway. Mol Ther Nucleic Acids.

[63] Wang Y, Yin L, Sun X (2020). CircRNA hsa_circ_0002577 accelerates endometrial cancer progression through activating IGF1R/PI3K/Akt pathway. J Exp Clin Cancer Res.

[64] Shatsky IN, Terenin IM, Smirnova VV, Andreev DE (2018). Cap-independent translation: what’s in a name?. Trends Biochem Sci.

[65] Hinnebusch AG (2014). The scanning mechanism of eukaryotic translation initiation. Annu Rev Biochem.

[66] Sonenberg N, Hinnebusch AG (2009). Regulation of translation initiation in eukaryotes: mechanisms and biological targets. Cell.

[67] Merrick WC, Pavitt GD (2018). Protein synthesis initiation in eukaryotic cells. Cold Spring Harb Perspect Biol.

[68] Borden KLB, Volpon L (2020). The diversity, plasticity, and adaptability of cap-dependent translation initiation and the associated machinery. RNA Biol.

[69] Smith RCL, Kanellos G, Vlahov N, Alexandrou C, Willis AE, Knight JRP (2021). Translation initiation in cancer at a glance. J Cell Sci.

[70] Kwan T, Thompson SR (2019). Noncanonical translation initiation in eukaryotes. Cold Spring Harb Perspect Biol.

[71] Godet AC, David F, Hantelys F, Tatin F, Lacazette E, Garmy-Susini B (2019). IRES trans-acting factors, key actors of the stress response. Int J Mol Sci.

[72] Jang SK, Kräusslich HG, Nicklin MJ, Duke GM, Palmenberg AC, Wimmer E (1988). A segment of the 5’ nontranslated region of encephalomyocarditis virus RNA directs internal entry of ribosomes during in vitro translation. J Virol.

[73] Han S, Wang X, Guan J, Wu J, Zhang Y, Li P (2021). Nucleolin promotes IRES-driven translation of foot-and-mouth disease virus by supporting the assembly of translation initiation complexes. J Virol.

[74] Lang KJ, Kappel A, Goodall GJ (2002). Hypoxia-inducible factor-1alpha mRNA contains an internal ribosome entry site that allows efficient translation during normoxia and hypoxia. Mol Biol Cell.

[75] Shatsky IN, Dmitriev SE, Terenin IM, Andreev DE (2010). Cap- and IRES-independent scanning mechanism of translation initiation as an alternative to the concept of cellular IRESs. Mol Cells.

[76] Faye MD, Holcik M (2015). The role of IRES trans-acting factors in carcinogenesis. Biochim Biophys Acta.

[77] Holcik M, Sonenberg N (2005). Translational control in stress and apoptosis. Nat Rev Mol Cell Biol.

[78] Weingarten-Gabbay S, Elias-Kirma S, Nir R, Gritsenko AA, Stern-Ginossar N, Yakhini Z (2016). Comparative genetics. Systematic discovery of cap-independent translation sequences in human and viral genomes. Science.

[79] Chen X, Han P, Zhou T, Guo X, Song X, Li Y (2016). circRNADb: a comprehensive database for human circular RNAs with protein-coding annotations. Sci Rep.

[80] Yang Y, Wang Z (2019). IRES-mediated cap-independent translation, a path leading to hidden proteome. J Mol Cell Biol.

[81] Wang Y, Wang Z (2015). Efficient backsplicing produces translatable circular mRNAs. RNA.

[82] Lamphear BJ, Kirchweger R, Skern T, Rhoads RE (1995). Mapping of functional domains in eukaryotic protein synthesis initiation factor 4G (eIF4G) with picornaviral proteases. Implications for cap-dependent and cap-independent translational initiation. J Biol Chem.

[83] Haizel SA, Bhardwaj U, Gonzalez RL, Mitra S, Goss DJ (2020). 5’-UTR recruitment of the translation initiation factor eIF4GI or DAP5 drives cap-independent translation of a subset of human mRNAs. J Biol Chem.

[84] Stoneley M, Willis AE (2004). Cellular internal ribosome entry segments: structures, trans-acting factors and regulation of gene expression. Oncogene.

[85] King HA, Cobbold LC, Willis AE (2010). The role of IRES trans-acting factors in regulating translation initiation. Biochem Soc Trans.

[86] Li S, Mason CE (2014). The pivotal regulatory landscape of RNA modifications. Annu Rev Genomics Hum Genet.

[87] Csepany T, Lin A, Baldick CJ, Beemon K (1990). Sequence specificity of mRNA N6-adenosine methyltransferase. J Biol Chem.

[88] Ping XL, Sun BF, Wang L, Xiao W, Yang X, Wang WJ (2014). Mammalian WTAP is a regulatory subunit of the RNA N6-methyladenosine methyltransferase. Cell Res.

[89] Garcias Morales D, Reyes JL (2021). A birds’-eye view of the activity and specificity of the mRNA m(6) A methyltransferase complex. Wiley Interdiscip Rev RNA.

[90] Jia G, Fu Y, Zhao X, Dai Q, Zheng G, Yang Y (2011). N6-methyladenosine in nuclear RNA is a major substrate of the obesity-associated FTO. Nat Chem Biol.

[91] Zheng G, Dahl JA, Niu Y, Fedorcsak P, Huang CM, Li CJ (2013). ALKBH5 is a mammalian RNA demethylase that impacts RNA metabolism and mouse fertility. Mol Cell.

[92] Wang T, Kong S, Tao M, Ju S (2020). The potential role of RNA N6-methyladenosine in cancer progression. Mol Cancer.

[93] Liu N, Dai Q, Zheng G, He C, Parisien M, Pan T (2015). N(6)-methyladenosine-dependent RNA structural switches regulate RNA-protein interactions. Nature.

[94] Zhou C, Molinie B, Daneshvar K, Pondick JV, Wang J, Van Wittenberghe N (2017). Genome-wide maps of m6A circRNAs identify widespread and cell-type-specific methylation patterns that are distinct from mRNAs. Cell Rep.

[95] Meyer KD, Patil DP, Zhou J, Zinoviev A, Skabkin MA, Elemento O (2015). 5’ UTR m(6)A promotes cap-independent translation. Cell.

[96] Kong S, Tao M, Shen X, Ju S (2020). Translatable circRNAs and lncRNAs: driving mechanisms and functions of their translation products. Cancer Lett.

[97] Shi Y, Jia X, Xu J (2020). The new function of circRNA: translation. Clin Transl Oncol.

[98] Abe N, Matsumoto K, Nishihara M, Nakano Y, Shibata A, Maruyama H (2015). Rolling circle translation of circular RNA in living human cells. Sci Rep.

[99] Perriman R, Ares M (1998). Circular mRNA can direct translation of extremely long repeating-sequence proteins in vivo. RNA.

[100] Liu Y, Li Z, Zhang M, Zhou H, Wu X, Zhong J (2021). Rolling-translated EGFR variants sustain EGFR signaling and promote glioblastoma tumorigenicity. Neuro Oncol.

[101] Wawrzyniak O, Zarębska Ż, Kuczyński K, Gotz-Więckowska A, Rolle K (2020). Protein-related circular RNAs in human pathologies. Cells.

[102] AbouHaidar MG, Venkataraman S, Golshani A, Liu B, Ahmad T (2014). Novel coding, translation, and gene expression of a replicating covalently closed circular RNA of 220 nt. Proc Natl Acad Sci U S A.

[103] Liu J, Gough J, Rost B (2006). Distinguishing protein-coding from non-coding RNAs through support vector machines. PLoS Genet.

[104] Sun L, Luo H, Bu D, Zhao G, Yu K, Zhang C (2013). Utilizing sequence intrinsic composition to classify protein-coding and long non-coding transcripts. Nucleic Acids Res.

[105] Aspden JL, Eyre-Walker YC, Phillips RJ, Amin U, Mumtaz MA, Brocard M (2014). Extensive translation of small Open Reading Frames revealed by Poly-Ribo-Seq. Elife.

[106] Galindo MI, Pueyo JI, Fouix S, Bishop SA, Couso JP (2007). Peptides encoded by short ORFs control development and define a new eukaryotic gene family. PLoS Biol.

[107] Andrews SJ, Rothnagel JA (2014). Emerging evidence for functional peptides encoded by short open reading frames. Nat Rev Genet.

[108] Huang W, Ling Y, Zhang S, Xia Q, Cao R, Fan X (2021). TransCirc: an interactive database for translatable circular RNAs based on multi-omics evidence. Nucleic Acids Res.

[109] Li H, Xie M, Wang Y, Yang L, Xie Z, Wang H (2021). riboCIRC: a comprehensive database of translatable circRNAs. Genome Biol.

[110] Brar GA, Weissman JS (2015). Ribosome profiling reveals the what, when, where and how of protein synthesis. Nat Rev Mol Cell Biol.

[111] Ingolia NT, Hussmann JA, Weissman JS (2019). Ribosome profiling: global views of translation. Cold Spring Harb Perspect Biol.

[112] Andreev DE, O’Connor PB, Loughran G, Dmitriev SE, Baranov PV, Shatsky IN (2017). Insights into the mechanisms of eukaryotic translation gained with ribosome profiling. Nucleic Acids Res.

[113] Chassé H, Boulben S, Costache V, Cormier P, Morales J (2017). Analysis of translation using polysome profiling. Nucleic Acids Res.

[114] Pringle ES, McCormick C, Cheng Z (2019). Polysome profiling analysis of mRNA and associated proteins engaged in translation. Curr Protoc Mol Biol.

[115] Domon B, Aebersold R (2006). Mass spectrometry and protein analysis. Science.

[116] Housman G, Ulitsky I (2016). Methods for distinguishing between protein-coding and long noncoding RNAs and the elusive biological purpose of translation of long noncoding RNAs. Biochim Biophys Acta.

[117] Zhao H, Zhou Q, Li X (2021). Protein bait hypothesis: circRNA-encoded proteins competitively inhibit cognate functional isoforms. Trends Genet.

[118] Rybak-Wolf A, Stottmeister C, Glažar P, Jens M, Pino N, Giusti S (2015). Circular RNAs in the mammalian brain are highly abundant, conserved, and dynamically expressed. Mol Cell.

[119] Yang Y, Gao X, Zhang M, Yan S, Sun C, Xiao F (2018). Novel role of FBXW7 circular RNA in repressing glioma tumorigenesis. J Natl Cancer Inst.

[120] Zhang M, Huang N, Yang X, Luo J, Yan S, Xiao F (2018). A novel protein encoded by the circular form of the SHPRH gene suppresses glioma tumorigenesis. Oncogene.

[121] Xia X, Li X, Li F, Wu X, Zhang M, Zhou H (2019). A novel tumor suppressor protein encoded by circular AKT3 RNA inhibits glioblastoma tumorigenicity by competing with active phosphoinositide-dependent Kinase-1. Mol Cancer.

[122] Zhang M, Zhao K, Xu X, Yang Y, Yan S, Wei P (2018). A peptide encoded by circular form of LINC-PINT suppresses oncogenic transcriptional elongation in glioblastoma. Nat Commun.

[123] Gao X, Xia X, Li F, Zhang M, Zhou H, Wu X (2021). Circular RNA-encoded oncogenic E-cadherin variant promotes glioblastoma tumorigenicity through activation of EGFR-STAT3 signalling. Nat Cell Biol.

[124] Wu X, Xiao S, Zhang M, Yang L, Zhong J, Li B (2021). A novel protein encoded by circular SMO RNA is essential for Hedgehog signaling activation and glioblastoma tumorigenicity. Genome Biol.

[125] Zheng X, Chen L, Zhou Y, Wang Q, Zheng Z, Xu B (2019). A novel protein encoded by a circular RNA circPPP1R12A promotes tumor pathogenesis and metastasis of colon cancer via Hippo-YAP signaling. Mol Cancer.

[126] Pan Z, Cai J, Lin J, Zhou H, Peng J, Liang J (2020). A novel protein encoded by circFNDC3B inhibits tumor progression and EMT through regulating Snail in colon cancer. Mol Cancer.

[127] Zhi X, Zhang J, Cheng Z, Bian L, Qin J. circLgr4 drives colorectal tumorigenesis and invasion through Lgr4-targeting peptide. Int J Cancer. 2019. 10.1002/ijc.32549. 10.1002/ijc.32549 31269234

[128] Ye F, Gao G, Zou Y, Zheng S, Zhang L, Ou X (2019). circFBXW7 inhibits malignant progression by sponging miR-197-3p and encoding a 185-aa protein in triple-negative breast cancer. Mol Ther Nucleic Acids.

[129] Li J, Ma M, Yang X, Zhang M, Luo J, Zhou H (2020). Circular HER2 RNA positive triple negative breast cancer is sensitive to Pertuzumab. Mol Cancer.

[130] Li Y, Chen B, Zhao J, Li Q, Chen S, Guo T (2021). HNRNPL circularizes ARHGAP35 to produce an oncogenic protein. Adv Sci (Weinh).

[131] Liang WC, Wong CW, Liang PP, Shi M, Cao Y, Rao ST (2019). Translation of the circular RNA circβ-catenin promotes liver cancer cell growth through activation of the Wnt pathway. Genome Biol.

[132] Jiang T, Xia Y, Lv J, Li B, Li Y, Wang S (2021). A novel protein encoded by circMAPK1 inhibits progression of gastric cancer by suppressing activation of MAPK signaling. Mol Cancer.

[133] Lewis TS, Shapiro PS, Ahn NG (1998). Signal transduction through MAP kinase cascades. Adv Cancer Res.

